# iTRAQ-Based Proteomic Analysis Reveals Potential Regulation Networks of IBA-Induced Adventitious Root Formation in Apple

**DOI:** 10.3390/ijms19030667

**Published:** 2018-02-27

**Authors:** Chao Lei, Sheng Fan, Ke Li, Yuan Meng, Jiangping Mao, Mingyu Han, Caiping Zhao, Lu Bao, Dong Zhang

**Affiliations:** College of Horticulture, Northwest A&F University, Yangling 712100, China; lfeiyuc@nwafu.edu.cn (C.L.); fancier@nwafu.edu.cn (S.F.); keli505@nwafu.edu.cn (K.L.); myhh0430@nwafu.edu.cn (Y.M.); mjp588@nwafu.edu.cn (J.M.); hanmy@nwsuaf.edu.cn (M.H.); zhcc@nwsuaf.edu.cn (C.Z.); baolu@nwsuaf.edu.cn (L.B.)

**Keywords:** Isobaric tags for relative and absolute quantification (iTRAQ), apple rootstock, Indole-3-butyric acid (IBA), adventitious root formation

## Abstract

Adventitious root (AR) formation, which is controlled by endogenous and environmental factors, is indispensable for vegetative asexual propagation. However, comprehensive proteomic data on AR formation are still lacking. The aim of this work was to study indole-3-butyric acid (IBA)-induced AR formation in the dwarf apple rootstock ‘T337’. In this study, the effect of IBA on AR formation was analysed. Subsequent to treatment with IBA, both the rooting rate and root length of ‘T337’ increased significantly. An assessment of hormone levels in basal stem cuttings suggested that auxin, abscisic acid, and brassinolide were higher in basal stem cuttings that received the exogenous IBA application; while zeatin riboside, gibberellins, and jasmonic acid were lower than non-treated basal stem cuttings. To explore the underlying molecular mechanism, an isobaric tags for relative and absolute quantification (iTRAQ)-based proteomic technique was employed to identify the expression profiles of proteins at a key period of adventitious root induction (three days after IBA treatment). In total, 3355 differentially expressed proteins (DEPs) were identified. Many DEPs were closely related to carbohydrate metabolism and energy production, protein homeostasis, reactive oxygen and nitric oxide signaling, and cell wall remodeling biological processes; as well as the phytohormone signaling, which was the most critical process in response to IBA treatment. Further, RT-qPCR analysis was used to evaluate the expression level of nine genes that are involved in phytohormone signaling and their transcriptional levels were mostly in accordance with the protein patterns. Finally, a putative work model was proposed. Our study establishes a foundation for further research and sheds light on IBA-mediated AR formation in apple as well as other fruit rootstock cuttings.

## 1. Introduction

Apple (*Malus* x *domestica* Borkh.) is one of the most planted and utilized tree fruits in the world. Apple fruits have great nutritional value and are of significant economic importance. In recent decades, the use of dwarfing apple rootstocks has been dominant in the production of apples worldwide. As a consequent, there has been significantly increased demand for the breeding of dwarfing apple rootstocks. Asexual reproduction is widely used for rootstock breeding due to its high efficiency, short cycle, and maintenance of genetic stability. The induction of adventitious roots (ARs) from basal stem cuttings is a key step in the asexual reproduction of apple rootstocks. The molecular mechanisms underlying adventitious rooting, however, are still not completely understood. High-quality apple rootstocks play an important role in regulating the environmental adaptability of apple trees. ‘T337’, is a cultivar of apple dwarfing rootstock that is characterized by strong growth control, induction of early fruiting, high yields, and the production of high-quality apples from grafted scion cultivars. Thus, the study of the molecular regulatory mechanisms underlying AR formation in ‘T337’ apple rootstock has important theoretical and practical value for apple rootstock breeding.

ARs are post-embryonic roots that arise from leaves, stems, and non-pericycle tissue in older roots, which differs from lateral roots (LRs) that emerges from primary roots within the pericycle [1]. Although ARs and LRs do share some similarities, they also have differences to one another [2]. Two patterns of ARs formation, direct and indirect, have been previously reported. In the direct pattern, root primordia directly emerge from established cell types, such as the cambium and vascular tissues. In the indirect pattern, even if the same tissues often take part, callus tissue is produced prior to the formation of root primordia [1]. A previous study also reported that AR formation is always divided into three phases: induction, initiation, and expression [3]. During the AR induction process, plants necessarily perceive a stimulus to reprogram target cells to form meristemoid cells, but without the occurrence of any notable level of cell division. Dediffierentation of target cells in apple takes place prior to the induction phase [4]. An AR primordium is formed during the initiation phase by several rounds of periclinal and tangential division in cells of the previously mentioned tissues. The AR expression process is characterized by growth of the AR primordium and root emergence. 

AR formation is a complex process that can be affected by numerous factors, both internal and external, such as sugars, phytohormones, mineral salts, light conditions, wounding, waterlogging, and temperature [1]. In particular, phytohormones play a crucial role in the control of AR formation as hormone levels respond to changes in the environment, provide a signaling network within the plant, and play a decisive role in cell fate determination and specialization. Auxin, mainly indole-3-acetic acid (IAA), is the principal phytohormone that is responsible for initiating rooting and serves a critical role in the first phases of AR development [5]. The auxin response factors ARF6 and ARF8 have been reported as the major players that mediate auxin signaling during AR formation, while ARF17 has an opposite effect [6]. In *Arabidopsis*, IAA induces AR formation by inducing the influx (Auxin Resistant 1/Like AUX—AUX/LAX) and efflux (PIN-Formed—PIN) carriers to establish an auxin gradient, which is essential for AR induction [5,7]. An ATP-binding cassette transporter, ABCB19, was also demonstrated to be associated with the auxin efflux associated with AR formation [8]. Exogenous application of indole-3-butyric acid (IBA), which is a natural precursor of IAA [9], has been widely used recently in rootstock propagation because it is more stable and effective than IAA in promoting AR development, largely due to its higher resistance to light-induced degradation [10,11,12,13,14]. Although the exact mechanism of IBA action has not yet been demonstrated, some assumptions have been established. One supposition is that IBA serves as a storage form of auxin and is inactive during its cell-to-cell transit until it is converted to IAA in target cells [15]. Other evidence suggests, however, that IBA itself might serve as an auxin and does not act via the conversion to IAA [13].

Apart from auxin, a large body of evidence suggests that other plant hormones also modulate the formation of ARs [16,17,18]. For example, cytokinins (CKs) were shown to be antagonistic to auxin and suppress rather than promote AR induction in many species, including Arabidopsis, rice, and poplar [16,19,20]. Exogenous gibberellins (GA) inhibited AR formation and rice mutants that are deficient in GA biosynthesis produced a greater number of ARs [18]. Furthermore, other less predominant processes are also important in AR induction, such as water balance, stress response, carbohydrate sink establishment, and cell wall degradation and assembly [21]. In general, despite the importance of many factors in AR formation that have been reported in numerous studies, the molecular mechanisms underlying AR formation need to be further detailed, especially in non-model species, such as apple. 

Due to its ability to obtain quantitative measurements of complex proteomic changes in tissue samples, an isobaric tag for a relative and absolute quantitation (iTRAQ)-based liquid chromatograph-mass spectrometer/mass spectrometer (LC-MS/MS) proteomic analysis has been widely used to study plant growth and development processes, abiotic and biotic stress responses, fruit ripening, and quality, etc. [22,23,24]. Proteomic technologies have been employed in the study of AR formation in several species, including *cucumber* [25] and *Arabidopsis* [26]. Detailed proteomic studies of AR formation in apple rootstock, however, have not been reported. 

In the current study, iTRAQ-based quantitative proteomic analysis was employed to characterize the proteins that are involved in the process of IBA-induced AR formation in ‘T337’ apple rootstocks. Differentially expressed proteins (DEPs) in basal stem cuttings of ‘T337’ at three days after IBA treatment and non-treated controls were identified and quantified. To elucidate the function of the DEPs in the induction phase of AR formation, the pathways and functional roles of the identified proteins were analyzed, such as phytohormone signaling, carbohydrate metabolism, and energy production, etc. The results of the study provided important insights into the underlying molecular mechanism of AR induction in apple rootstocks. 

## 2. Results

### 2.1. Effects of Exogenous IBA on AR Formation

According to previous anatomical studies in apple [4,27], basal stem samples treated with 1 mg·L^−1^ IBA and non-treated control were harvested at five key time points 0, 3, 7, 11, and 16 days after the treatment with IBA. AR formation was divided into four stages based on morphological changes: stage 1 represents cuttings just treated with IBA (0 day, Competent cells), stage 2 represents cuttings in the AR induction phase (3 day, Cell cycle reactivation), stage 3 represents cuttings in the AR initiation phase (7 day and 11 day, Activation of the callus formation and AR primordium), and stage 4 represents cuttings where ARs have broken through the epidermis and emerged (16 day, AR outgrowth) [28,29]. Samples consisted of the basal parts of stems (approximately 0.5 cm of root zone); and were excised from the tissue culture cuttings. Proteins were extracted from samples collected at 3 day, which represents the critical AR induction period [28,29].

**Figure 1:**
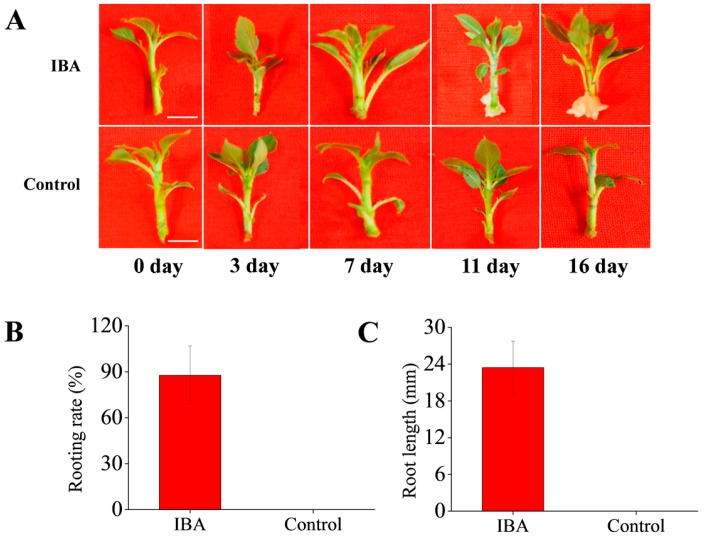
Effects of indole-3-butyric acid (IBA) on adventitious root formation in apple rootstock ‘T337’. (**A**) Morphological changes in ‘T337’ cultural cuttings after treatment with IBA and control for 0, 3, 7, 11, 16 days. Scale bars = 0.5 cm. (**B**) Rooting rate in ‘T337’ with IBA treatment and control at 16 days per 50 cuttings. (**C**) Root length in ‘T337’ with IBA treatment and control at 16 days per 50 cuttings.

Representative phenotypes during AR formation were recorded in ‘T337’ tissue culture seedlings in response to exogenous IBA treatment. As illustrated in Figure 1A, early signs of callus formation were observed at 7 day in IBA-treated cuttings and callus formation was evident at 11 day. After 16 day, ARs emergence was observed. However, there were no signs of AR formation during the entire sampling period in non-treated cuttings. In addition, the rooting rate and root length in the ‘T337’ tissue culture seedlings at 16 day suggested that IBA induced root initiation and growth, which could not occur without IBA treatment. (Figure 1B,C).

### 2.2. Hormonal Changes in ‘T337’ Basal Stem Cuttings during AR Formation

**Figure 2:**
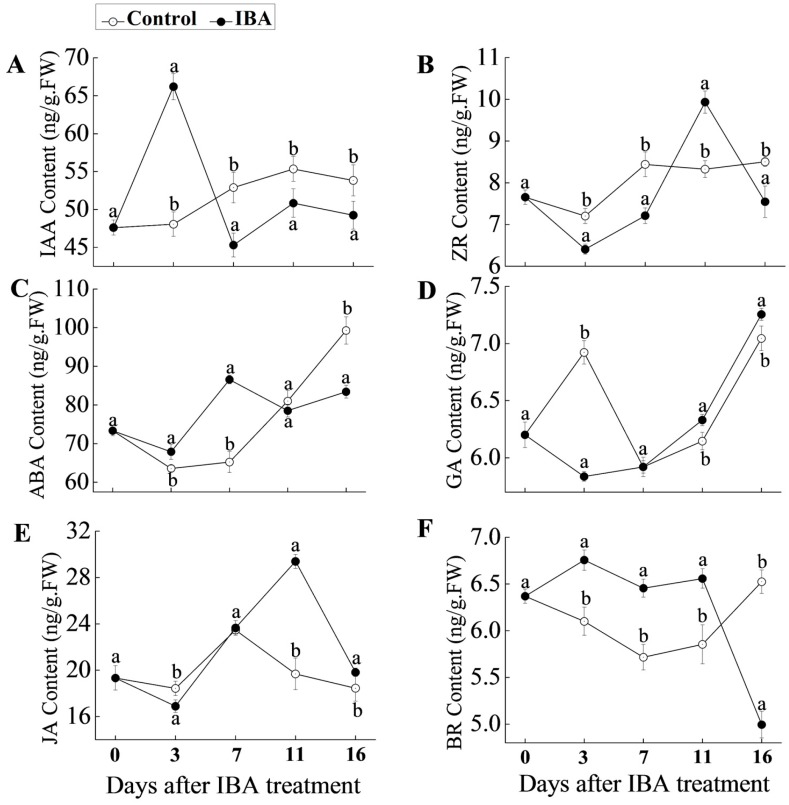
Endogenous hormone levels in ‘T337’ basal cuttings after treatment with IBA and control for 0, 3, 7, 11, 16 days. (**A**) Auxin (IAA) content; (**B**) Zeatin riboside (ZR) content; (**C**) Abscisic acid (ABA) content; (**D**) Gibberellins (GA) content; (**E**) Jasmonic acid (JA) content; and, (**F**) Brassinolide (BR) content. Statistically significant differences in IBA-treated cuttings and control on each day are indicated with ‘a’ and ‘b’. Data shows the average values ± SE of three independent experiments.

Since plant hormones play a crucial role in regulating AR formation, the abundance of six hormones was analyzed at five key time points of AR formation (Figure 2). Total indole-3-acetic acid (IAA) content significantly increased at 3 day in the IBA-treated plants, but then drastically decreased; falling below levels in control cuttings at 7 d and thereafter (Figure 2A). In relative comparison to the control, zeatin riboside (ZR) levels in the IBA-treated cuttings were reduced at 3, 7, and 16 day, and higher at 11 day (Figure 2B). Abscisic acid (ABA) levels exhibited a slight increase at 3 day in the IBA-treated samples and an obvious increase at 7 day d, but then decreased at 11 and 16 day (Figure 2C). Gibberellins (GA_1+3_) concentrations were initially lower in the IBA-treated samples at 3 day and then were similar to the control cuttings at 7 day, followed by higher levels in the IBA-treated cuttings (Figure 2D). Similar data were obtained for jasmonic acid (JA) content (Figure 2E). Lastly, brassinolide (BR) levels were consistently higher in the IBA-treated cuttings than in the controls at all time points, with the exception of 16 day (Figure 2F).

### 2.3. Potential Protein Identification

**Figure 3:**
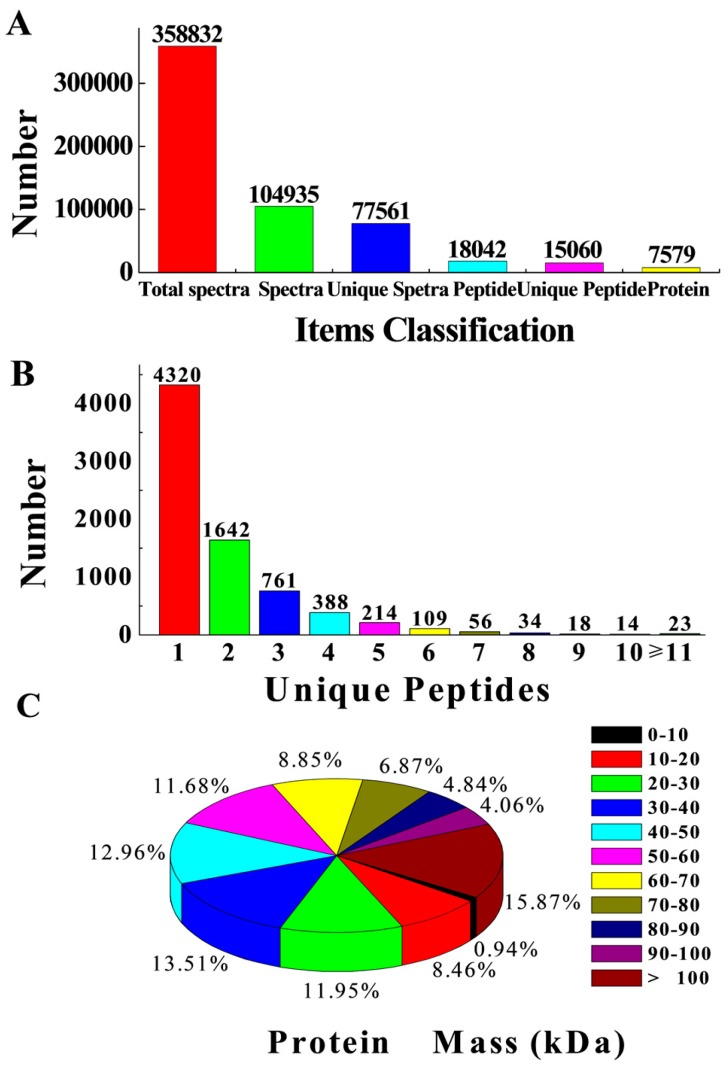
Results of the isobaric tag for a relative and absolute quantitation (iTRAQ) liquid chromatography/tandem mass spectrometry analysis of ‘T337’ basal cuttings with IBA treatment and control. (**A**) Identified proteins, Unique peptide: A protein-specific peptide; Unique spectra: spectra that matched unique peptides; (**B**) Number of unique peptides that were matched to proteins. The *X*-axis shows the unique peptide number of each protein, and the *Y*-axis shows the corresponding protein number; (**C**) Distribution of average molecular mass of identified proteins.

The gel-free iTRAQ system was utilized to analyze proteomic changes during the AR induction phase following the application of exogenous IBA. Expression profiles of proteins extracted from ‘T337’ basal stem cuttings after 3 d with IBA treatment were analyzed. A total of 358,832 spectra were generated, and 104,935 spectra were utilized after low-scoring spectra were eliminated (Table S1). Ultimately, 18,042 peptides, 15,060 unique peptides, and 7579 proteins were identified. (Figure 3A and Table S2). The distribution of unique peptides defining each protein is shown in Figure 3B, with over 43% of them, including at least two unique peptides (Table S2). The average molecular mass of the identified gene products was between 20 and 70 kDa (Figure 3C). The variation of the two biological replicates of each group (IBA-treated and untreated controls) was calculated according to their quantitative data, with most proteins exhibiting less than 20% variation (Figure S1), indicating high quality and repeatability of the data.

In total, 3355 differentially expressed proteins (DEPs) were identified. Among the identified DEPs, 1501 proteins were up-regulated (Table S3) and 1854 proteins were down-regulated (Table S4) in the IBA-treated cuttings, as compared with untreated controls. DEPs were identified as being differentially expressed when the fold changes ≥1.2 (the average of all comparison group ratios), and a *p*-value < 0.05 (Figure S2). 

### 2.4. Classification of Identified Proteins

**Figure 4:**
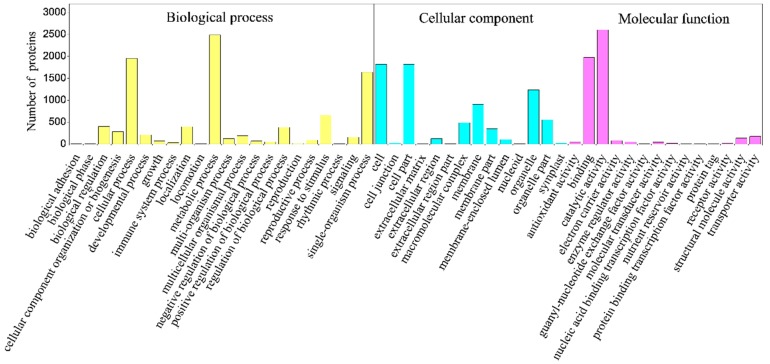
Gene Ontology (GO) analysis of differently expressed proteins (DEPs) in IBA-treated apple cuttings, compared with untreated controls. Expressed proteins involved in molecular function, cellular component and biological process against the GO database. The *X* axis represents the Gene Ontology functional classification; The *Y* axis represents the number of differentially expressed proteins.

The DEPs were individually analyzed against the Gene Ontology (GO) database using three sets of ontologies: biological process, molecular function, and cellular component. The most abundant proteins in the biological process category were related to metabolic process, cellular process, and single-organism process, followed by proteins that are responsive to stimulus and those involved in biological regulation. In the molecular function category, catalytic activity was the most prominent, followed by binding, transporter activity, structural molecular activity, and other activities. In the cellular component category, proteins were mainly associated with cell, cell part, organelle, and membrane and organelle part (Figure 4).

Cluster of Orthologous Groups of proteins (COG) analysis clustered the proteins into 24 clusters (Figure S3). Posttranslational modification, protein turnover, and chaperones contained the greatest number of identified proteins, followed by replication, recombination, repair, transcription, signal transduction mechanisms, and carbohydrate transport and metabolism. 

Kyoto Encyclopedia of Genes and Genomes (KEGG) pathway mapping, based on KEGG orthology terms for assignment, was also conducted to obtain further information about the potential functional role of the DEPs in the IBA treatment. Only significantly enriched pathway categories that had a *p*-value of lower than 0.05 were examined. Results indicated that the DEPs were mainly enriched into plant biology processes, including metabolic pathways, biosynthesis of secondary metabolites, starch and sucrose metabolism, ribosome, phenylpropanoid biosynthesis, pentose and glucuronate interconversions, peroxisome, carbon fixation in photosynthetic organisms and glycine, serine, and threonine metabolism (Table 1).

In order to gain further insight into the biological processes that respond to IBA, proteins that had a ratio ≤0.67 and ≥1.5, and *p*-value < 0.05, were selected for further analysis. In addition, we only focused on proteins related to phytohormone signaling, carbohydrate metabolism and energy production, protein homeostasis, and other functions (Table 2).

### 2.5. RT-qPCR Analysis

**Figure 5:**
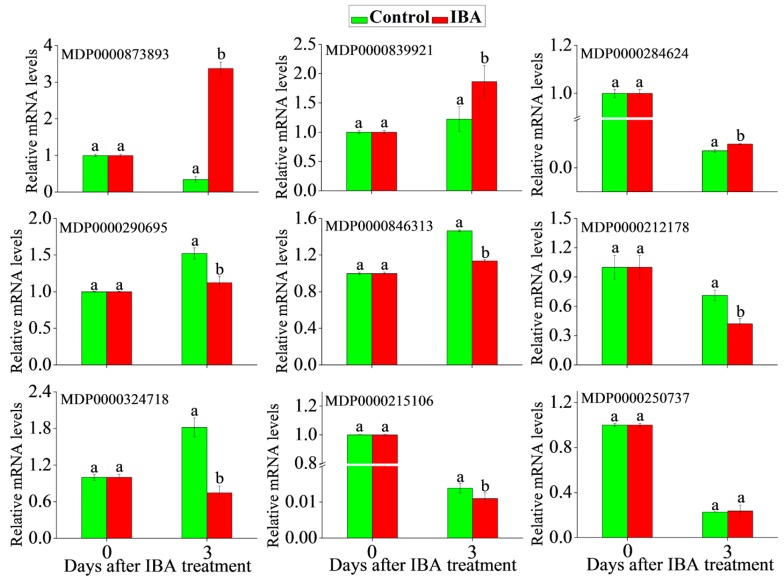
Analysis of change in mRNA level via qRT-PCR for several differential proteins mapped in phytohormone signaling pathway. The transcript levels at 0 d were normalized to value 1. Statistically significant differences in IBA-treated cuttings and control on each day were indicated with ‘a’ and ‘b’. Indole-3-acetic acid-amido synthetase (MDP0000873893), ACC oxidases (MDP0000839921), abscisic acid receptor PYL9 (MDP0000284624), indole-3-acetate *O*-methyltransferase 1 (MDP0000290695), two-component response regulator ARR1 (MDP0000846313), two-component response regulator ARR5 (MDP0000212178), ethylene-responsive transcription factor 4 (MDP0000324718), ABSCISIC ACID-INSENSITIVE 5-like protein (MDP0000215106), two-component response regulator ARR3 (MDP0000250737). Data shows the average values ± SE of three independent experiments. Letters above the bars indicate a statistically significant difference (*p* < 0.05), according to two-tailed *t*-test.

To obtain complementary information to the iTRAQ data, mRNA expressions levels were analyzed by RT-qPCR of nine genes related to phytohormone signaling. RNA of IBA-treated cuttings and controls were extracted at 0 and 3 day after IBA treatment, followed by qPCR analysis. For proteins that were in greater abundance in IBA-treated cuttings after 3 day IBA treatment, including indole-3-acetic acid-amido synthetase (MDP0000873893), ACC oxidases (MDP0000839921), and abscisic acid receptor PYL9 (MDP0000284624), all of them showed higher transcript abundances after 3 day IBA treatment. For the six selected proteins that were down-regulated in IBA-treated cuttings after 3 day IBA treatment, indole-3-acetate *O*-methyltransferase 1 (MDP0000290695), two-component response regulator ARR1 (MDP0000846313), two-component response regulator ARR5 (MDP0000212178), ethylene-responsive transcription factor 4 (MDP0000324718), and ABSCISIC ACID-INSENSITIVE 5-like protein (MDP0000215106) had lower transcript abundance as expected at 3 d while the transcript level of two-component response regulator ARR3 (MDP0000250737) differed from the obtained protein level (Figure 5). The discrepancy between the expression level of ARR3 and mRNA expression levels may be due to numerous factors, such as translational or post-translational regulation [30].

## 3. Discussion

AR formation, as an essential aspect of vegetative propagation, is a complex process requiring changes in the expression of a series of genes or proteins that induce the formation of new roots from stem cuttings or other vegetative tissues. The induction of ARs by the exogenous application of IBA has been reported in many species [11,12,13]. The underlying mechanism that is responsible for AR formation has not been comprehensively elucidated, especially at the level of the proteome. Therefore, an iTRAQ-based quantitative proteomic approach was used to provide new information pertaining to the induction phase (three days after IBA treatment) of IBA-induced AR formation. The discussion will focus on proteins related to phytohormone signaling, carbohydrate metabolism and energy production, reactive oxygen species (ROS) and nitric oxide (NO) signaling, protein homeostasis, and cell structure.

### 3.1. IBA May Regulate Phytohormone Signaling to Contribute to AR Induction

Auxins stimulate cells to produce meristemoids during the AR induction phase that have rhizogenic potential [31]. The growth of AR primordia, however, is independent of, and can even be inhibited by, auxins. A previous study on vine cuttings demonstrated that the level of endogenous auxin transiently increases during the AR induction phase. Although the lowest level of auxin occurs in the AR initiation phase, the level then begins to increase during the AR expression phase [32]. In our study, the level of total IAA exhibited a highly significant increase during the AR induction phase (3 days after IBA treatment) in IBA-treated cuttings, as compared with non-treated controls (Figure 2A). Regarding the increase in IAA content, many proteins related to IAA homeostasis and polar transport were identified in our data (Table 2). IAA is known to conjugate to amino acids, sugars, and peptides, all of which form a large part of the cellular pool of IAA [33]. Only free IAA, however, is deemed to be directly active, while its conjugates contribute to IAA homeostasis by inactivating IAA or serving as a reservoir of IAA that can be released by hydrolysis [34]. Indole-3-acetic acid-amido synthetases (MDP0000873893, MDP0000121609; GH3) and IAA carboxyl methyltransferase 1 (MDP0000290695; IAMT1) have been demonstrated to convert IAA to inactive forms [34,35]. In contrast, IAA-amino acid hydrolases (MDP0000310711, MDP0000663451; ILR1) functions in the hydrolysis of IAA-conjugates in plant cells to activate auxin signaling [33]. In our data, GH3 and ILR1 were up-regulated and IAMT1 showed a low abundance. This result showed that the shift between different forms of auxin plays an important role in maintaining auxin levels during the adventitious root induction phase. Members of the PINFORMED (PIN) protein family play a crucial role in auxin efflux and the subcellular localization of PIN determines the direction of auxin transport [21]. It is thought that the localization of PIN1 results from rapid actin-dependent cycling between the plasma membrane and endosomal compartments, which is influenced by vesicular trafficking [21]. Adenosine diphosphate (ADP)-ribosylation factor (Arf), a type of small guanosine triphosphate (GTP)-binding protein, is a core factor in vesicular trafficking [36]. In our study, three ADP-ribosylation factor GTPase-activating proteins (Arf GAPs) (MDP0000140463, MDP0000250432, and MDP0000288128) that regulates Arfs by converting the active GTP-bound forms of these proteins into their inactive guanosine diphosphate (GDP)-bound forms [37], were down-regulated in IBA-treated cuttings, which we suggest might serve to maintain efficient vesicular trafficking. 

Cytokinins (CKs) appear to be an inhibitor of AR induction and play an antagonistic role to auxin in more than one phase of AR formation [38]. Indeed, our results showed that IBA-treated samples displayed low zeatin riboside (ZR) (a type of CK) levels during AR induction phase (3 day after IBA treatment) (Figure 2B). A histidine-containing phosphotransfer protein 3 (MDP0000186518; AHP), a type-B two-component response regulator (MDP0000846313; ARR1) and three type-A two-component response regulators (MDP0000250737, ARR3; MDP0000212178, ARR5; MDP0000509768, ARR9) that are related to CK signal transduction, showed low levels (Table 2). CK signal transduction is based on a multi-step, two-component system (TCS), which utilizes AHP protein to transfer phosphate to B-type ARRs, thus inducing the transcription of type-A ARRs and others [39]. In *Arabidopsis*, ARR1 activated the expression of *short hypocotyl 2* (SHY2), which forms heterodimers with ARFs to negatively regulate *PIN* gene [40]. As a result, these data suggested that proteins related to CKs signaling might mediate the regulation of AR formation in IBA-treated apple samples.

Ethylene has recently been demonstrated to have a positive effect on the early induction and expression phases of AR formation and is strongly linked to the activity of auxin [41]. Application of the inhibitor of 1-aminocyclopropane-1-carboxylate synthase (ACS) and 1-aminocyclopropane-1-carboxylate synthase to de-rooted seedlings reduced and enhanced the number of ARs, respectively, clearly confirms that enhancing ethylene biosynthesis has a positive effect on AR formation [41]. In flood-induced AR formation in rice, ethylene signaling, rather than auxin, actives the cell cycle and reactive oxygen species (ROS) are subsequently produced, which play a beneficial role in the induction phase of AR formation [42]. In the present study, we identified four 1-aminocyclopropane-1-carboxylate (ACC) oxidases (MDP0000175691, MDP0000663852, MDP0000839921, MDP0000195885; ACO). Most of the ACO were highly abundant (Table 2); supporting a conclusion that ethylene biosynthesis was strongly stimulated at the levels of ACC synthesis and oxidation in IBA-treated samples. Many ethylene responsive transcription factors (ERFs) are continuously up-regulated during AR formation and are also induced in wounded leaves as well [41]. Ethylene-responsive transcription factor 4 (MDP0000324718; ERF4), which functions as a transcriptional repressor to modulate ethylene and abscisic acid responses [43], was down regulated. Collectively, these results demonstrated the important role of ethylene signaling during AR formation. 

Abscisic acid (ABA) content was slightly increased in IBA-treated cuttings at 3 d after treatment with IBA (Figure 2C). Indeed, a pyrabactin resistance-like protein 9 (MDP0000284624; PYL9), served as abscisic acid receptor, displayed increased abundance while an abscisic acid receptor PYL4 (MDP0000228470; PYL4) and an ABSCISIC ACID-INSENSITIVE 5-like protein (MDP0000215106; ABI5) exhibited low abundance in IBA-treated samples (Table 2). Higher abundance of PYLs can contact and inhibit protein phosphatases type-2C (PP2Cs), thus activating ABA signaling [44]. In addition, the overexpression of *ABI5* has relatively limited effects on enhancing ABA-responsive gene expression [45]. The result suggested the important role of ABA in AR induction in apple, which might relate to the role ABA plays in plant adaptation to stress [46].

Although none of the proteins identified in our proteomic analysis were related to gibberellins (GA), jasmonic acid (JA), and brassinolide (BR) homeostasis and signaling, hormone level data were obtained in this study. Previously, GA has been demonstrated to inhibit AR formation in *hybrid aspen*, as well as *Arabidopsis*. The inhibitory activity was shown to be linked to the perturbation of polar auxin transport, in particular auxin efflux in hybrid aspen, and both efflux and influx in Arabidopsis [47,48]. In our study, GA_1+3_ content significantly decreased in IBA-treated cuttings during the AR induction phase (3 days after IBA treatment) (Figure 2D), suggesting that GA may also have a negative effect on IBA-induced AR induction in apple. JA deficient mutants produced more ARs than wild-type plants [49,50]. JA levels are reduced by their conjugation with amino acids catalyzed by the expression of *GH3* auxin-induced genes, which results in an increase in the production of ARs [50]. In the current study, JA levels decreased in ‘T337’ apple cuttings in response to the IBA treatment at 3 day (Figure 2E), which corresponds to the inhibitory role of JA on AR induction. In contrast, however, BR, which has been reported to plays an antagonistic role in the inhibition of root growth by JA, were interestingly increased in response to IBA treatment at early AR phases (Figure 2F) [51]. 

### 3.2. Carbohydrate Metabolism and Energy Production may be Enhanced in Response to IBA 

During AR development, carbohydrates are an important energy and carbon skeleton source for cell divisions and the establishment of the new root meristems [52]. Low carbohydrate levels in cuttings inhibit the speed and quantity of AR development during the early phases of AR formation [53]. In relative comparison to light-grown controls, a dark treatment of cut hybrid *Petunia* stem segments resulted in increased carbohydrate levels in the basal portion of stems after they were transferred into the light. The increased level of carbohydrates increased and accelerated AR formation [54]. According to [52], the level of soluble sugars (glucose, fructose, sucrose) and starch increased after 72 h during AR formation. Here, an ADP-glucose pyrophosphorylase (MDP0000256619; AGPase) and a phosphoglucomutase (MDP0000866748; PGM) increased by >1.5-fold in IBA-treated samples relative to non-treated cuttings (Table 2). AGPase is regarded as the rate-limiting enzyme for starch synthesis due to its catalysis of the conversion of glucose-1-phosphate to ADP-glucose, which serves as a glucosyl donor for the elongation of α-1,4-glucosidic chains that are used in the synthesis of starch. PGM is crucial to glucose metabolism and functions in catalyzing the interconversion between glucose-1-phosphate and glucose-6-phosphate, helping to maintain a dynamic balance [55]. Our results indicated the importance for the role of carbohydrate metabolism in AR formation.

The tricarboxylic acid (TCA) cycle is an intermediate metabolic pathway for sugars, fats, and amino acids, involving the biosynthesis of amino acids and the formation of primary and secondary metabolites [56]. In our data, all of the protein species involved in the TCA cycle were clearly accumulated in IBA-treated samples (Table 2). Pyruvate dehydrogenase (MDP0000178814; PDH) catalyzes the oxidative decarboxylation of pyruvate, which serves as the central link connecting glycolysis with the TCA cycle [57]. Isocitrate dehydrogenase (MDP0000325085; ICDH) participates in the TCA cycle by catalyzing the interconversion between isocitric acid and 2-oxoglutarate. Additionally, 2-oxoglutarate (MDP0000214399; 2-OG) is converted to succinate dehydrogenase (SDH), which subsequently catalyzes the synthesis of succinate [58]. Arginine and aspartic acid are capable of binding to 2-oxoglutarate and ammonia to form glutamate, which serves as an important source of C and N [59]. Malic enzyme (MDP0000384593, MDP0000221561; MDH) can convert malate into oxaloacetic acid which is used in several metabolic pathways as a major source of a carbon skeleton and can also be reused in the TCA cycle. Collectively, these results suggest that the TCA cycle is significantly enhanced in IBA-treated cuttings and that it plays an essential role in AR induction. 

Glycolysis is a catabolic anaerobic pathway in which hexoses are oxidized to generate ATP, reductant, pyruvate, and building blocks for anabolism [60]. Proteins that are related to glycolysis were also significantly greater in IBA-treated cuttings, including a hexokinase (MDP0000823956, HXK), two 6-phosphofructokinases (MDP0000294262, MDP0000254412; PEK), three glyceraldehyde-3-phosphate dehydrogenases (MDP0000835914, MDP0000527995 and MDP0000543856; GAPDH), two 3-phosphoglycerate kinases (MDP0000174843, MDP0000212948; PGK), and an alcohol dehydrogenase (MDP0000236430; ADH) (Table 2). These findings are consistent with a previous report that AR formation in cucumber induced by waterlogging stress exhibited a high rate of glycolysis to overcome the energy crisis that is induced by the anaerobic conditions [25]. In addition, proteins related to the mitochondrial respiratory electron transport chain (ETC), including a nicotinamide adenine dinucleotide (NADH)-ubiquinone oxidoreductase 20 kDa subunit (MDP0000313179), a Cytochrome b-c1 complex subunit (MDP0000362465), and two Cytochrome c oxidases (MDP0000124616, MDP0000263444), were also increased (Table 2). The function of ETC is bio-oxidation, and coupled with ATP synthase, completes the process of oxidative phosphorylation and the production of ATP. This result suggested that oxidative phosphorylation was also enhanced, along with an increase in respiratory metabolism during the induction phase of AR formation. 

### 3.3. ROS and NO Signaling May Be Activated in Response to IBA 

The balance between reactive oxygen species (ROS) production and scavenging is indispensable to the regulation of cell death and growth. Research studies have demonstrated that ethylene-induced AR formation is regulated by ROS [61]. The inhibition of ROS production by NADPH oxidase, which inhibits the production of a major source (O^2−^) for ROS, also inhibited ethylene-induced AR formation [61]. Exogenous application of hydrogen peroxide (H_2_O_2_) as a rooting agent can take the place of auxin in olive cuttings [21]. Here, ten peroxidases (MDP0000319048, MDP0000545323, MDP0000243237, MDP0000208152, MDP0000154541, MDP0000192235, MDP0000706473, MDP0000283650, MDP0000136398, MDP0000301828, MDP0000209189; POD) were present at lower levels in IBA-treated samples (Table 2), which may result in the induction of H_2_O_2_ overproduction. Heat shock proteins (HSPs) are the most common ROS-related proteins and their induction has been shown to be linked to the presence of hydrogen peroxide [62]. Consistent with previous data in *cucumber* and *mung bean* [63,64], an increase in HSPs (MDP0000684170, MDP0000697285) has been proposed to act as a specific sensor of H_2_O_2_ levels in plants (Table 2) and may be the underlying basis for the higher level of H_2_O_2_ production in IBA-treated cuttings.

Nitric oxide (NO) has also been shown to be involved in AR formation in many species [65,66,67]. Based on a previous study in cucumber explants, proteins extracted form plants with NO-donor sodium nitroprusside (SNP) and IAA-treatment showed an activity of protein kinase, which could be inhibited by mitogen-activated protein kinases (MAPKs) inhibitor. Application of MAPK inhibitor to explants treated with SNP or IAA delayed root emergency and reduced AR numbers. This result indicated that a MAPK signaling cascade is activated during the adventitious root process mediated by NO [68]. In our iTRAQ data, a MAPK protein (MDP0000251955) significantly increased (Table 2). As a result, we speculated that MAPK-mediated NO signaling might participate in AR formation in apple, which needed to be further learnt.

### 3.4. IBA Treatment May Change Protein Homeostasis, Especially Ubiquitinylation-Based Protein Degradation

A vast number of studies have demonstrated that plants have exquisitely regulated protein degradation machinery [69,70]. Ubiquitinylation is the mechanism by which plants, and other organisms, flag proteins that are targeted to the proteasome for degradation [69,71]. Ubiquitinylation is a post-translational modification of proteins that is dependent upon a set of three enzymes, E1 (ubiquitin activating enzymes), E2 (ubiquitin conjugating enzymes), and E3 (ubiquitin ligase), in conjunction with the 26S proteasome complex [72]. In our study, three E3 ubiquitin ligases (MDP0000241084, MDP0000317971, MDP0000269081), two ATP-dependent 26S proteasome regulatory subunits (MDP0000676693, MDP0000322270), and two ubiquitin C-terminal hydrolases (MDP0000245541, MDP0000283283) that are involved in ubiquitinylation varied significantly between IBA-treated and untreated samples (Table 2), suggesting that ubiquitinylation plays an important role in the induction phase of AR formation in apple rootstocks. Additionally, two F-box proteins (MDP0000263256, MDP0000180936), which may be functionally related to phytohormone signaling, were dramatically up-regulated in IBA-treated cuttings. An F-box protein is one of the major components of the Skp1—Cul1—F-box protein (SCF) complex, which is a type of E3 ubiquitin ligase that can specifically recognize the ubiquitinated substrates [73]. Auxin signaling relies on the ‘molecular glue’ function of F-box proteins that strengthen the binding between SCF TIR1/AFB complexes and Aux/IAA proteins, resulting in the degradation of Aux/IAA by the 26S proteasome. Aux/IAA proteins are transcriptional repressors that act via dimerization with auxin-responsive transcription factors (ARFs). Consequently, the degradation of Aux/IAA protein releases the transcription activity of ARFs, thus allowing auxin-responsive genes to be expressed [74]. GA and JA signaling is also related to the identification and ubiquitination degradation of the transcriptional repressor, DELLA-domain protein and jasmonate ZIM-domain (JAZ) protein, respectively [75,76]. We speculate that exogenous IBA enhances ubiquitination, which may facilitate the degradation of proteins that are involved in phytohormone signal transduction during the induction phase of AR formation. 

Moreover, plenty of proteins related to protein synthesis and folding, such as ribosomal proteins, elongation factor, eukaryotic translation initiation factor, thiol-disulfide isomerase, and thioredoxins, were also differently expressed during AR induction phase in response to IBA treatment (Table 2). The differential regulation of proteins representing various components of the protein translation machinery suggests that there was a complex mechanism controlling protein synthesis during AR induction. Future detailed studies are warranted and necessary to further understand this phenomenon. 

### 3.5. The Effect of IBA on Microtubules and Cell Wall Properties

Microtubules (MTs) are one of three principal types of protein filaments that comprise the cytoskeleton in eukaryotic cells. Genes encoding MTs and MT-associated proteins play an important role in AR formation in carnation plant cuttings, where they are transiently down-regulated during the dedifferentiation phase and then up-regulated [77]. A recent study on *Eucalyptus grandis* cuttings indicated that MTs play a role in the shift from cell division to cell differentiation during AR induction [78]. In relative comparison to wild-type plants, fewer ARs formed in the temperature-sensitive mutant *mor1-1*, in which the MT-associated protein MOR1 was mutated; and, in the *bot1-1* mutant, where the MT-severing protein KATANIN is mutated. These mutants produced callus instead of dome-like AR primordia that are typically observed in wild-type plants. Detailed analysis of auxin transport, the organization of MTs, and cell wall properties indicates that fine-tuned crosstalk between MTs, cell walls, and auxin transport is crucial for the shift from cell division to cell differentiation during AR formation in *Arabidopsis* [78]. Here, the levels of tubulin α-3 (MDP0000812416) increased and an α-tubulin suppressor (MDP0000282827) was reduced in IBA-treated samples (Table 2). On the other hand, most of the proteins related to cell wall properties, including two xyloglucan endotransglucosylase/hydrolase proteins (MDP0000296747, MDP0000661960), an expansin-like protein (MDP0000640549), five pectin lyase-like superfamily proteins (MDP0000130769, MDP0000248311, MDP0000943790, MDP0000175757, MDP0000251956), a cellulase (MDP0000753366), and an α-l-arabinofuranosidase (MDP0000055078), had significantly high fold changes in IBA-treated samples. Collectively, these data suggested that the cell walls were undergoing modification [25,79], which can work with MTs-related proteins on AR induction phase in apple rootstocks. 

## 4. Materials and Methods

### 4.1. Plant Material 

The ‘T337’s apple rootstocks were derived from tissue culture and cultivated in an environmental chamber located in the Northwest Agriculture and Forestry University, Yangling (108°04′ E, 34°16′ N), China. The tissue culture cuttings were grown under a 16-h-light, 25°/8-h-dark, 15° cycle. Relative humidity was approximately 70–80%. White light was generated by three fluorescent tubes (FSL T8 36W/765, Foshan, Guangdong, China). Half of the ‘T337’ tissue culture cuttings were transferred into a rooting medium, containing 1/2MS, 20 g·L^−1^ sugar and 8 g L^−1^ agar and 1 mg·L^−1^ IBA. Control plants were transferred into similar media which lacked the presence of IBA.

In order to ensure the ability to conduct statistical analyses, two biological replicates (60 plants per replicate) were used for the iTRAQ-based quantitative proteomic analysis and three biological replicates (60 plants per replicate) were used for hormone measurements and RT-qPCR analysis [22]. Collected samples were immediately flash frozen in liquid nitrogen and stored at −80° until further use.

### 4.2. Measurement of Hormone Contents

The extraction and purification of total indole-3-acetic acid (IAA), zeatin riboside (ZR), abscisic acid (ABA), gibberellins (GA_1+3_), jasmonic acid (JA), and brassinolide (BR) were performed according to previously described methods [80]. Hormone levels were measured using a high-performance liquid chromatography system (Waters 2489 UV/Visible detector, Waters, Milford, Massachusetts, USA), which was linked to a Waters 1525 binary High Performance Liquid Chromatography (HPLC) pump and a Waters C_18_ column (4.6 mm × 250 mm, 5 μm). 10 μL of each sample was injected into an HPLC pump set at a flow rate of 0.6 mL·min^−1^ at 25 °C and the mobile phase consisted of a mixture of methanol/water/acetic acid (45:54.2:0.8, *v/v/v*). External pure standards of IAA, ZR, ABA, GA, and JA (Sigma, San Francisco, CA, USA) were used for quantification. The determination of BR was performed according to [81]. All of the measurements in this part were completed with three biological and technological replicates (200 mg per replicate). 

### 4.3. Protein Extraction

Samples that were harvested at 3 d after IBA treatment (approximately 500 mg fresh weight, from control and treatment groups with two biological replicates, respectively) were ground to a fine powder in liquid nitrogen extracted with lysis buffer (7 M urea, 2 M thiourea, 4% CHAPS, 40 mM Tris-HCl, pH 8.5) containing 1 mM PMSF (phenylmethylsulfonyl fluoride) and 2 mM EDTA (final concentration). The samples were vortexed and subsequently allowed to stand for 5 min. Afterwards, 10 mM DTT (final concentration) was added to each sample. The suspension was sonicated at 200 W for 15 min and then centrifuged at 4 °C, 30,000× *g* for 15 min. The supernatant was removed and mixed with 5× volume of chilled acetone containing 10% (*v/v*) TCA and incubated at −20 °C overnight. After centrifugation at 4 °C, 30,000× *g*, the supernatant was discarded. The precipitate was washed with chilled acetone three times. The remaining pellet was then air-dried and dissolved in lysis buffer (7 M urea, 2 M thiourea, 4% NP40, 20 mM Tris-HCl, pH 8.0–8.5). The suspension was sonicated at 200 W for 15 min and then centrifuged at 4 °C, 30,000× *g* for 15 min. The supernatant was then transferred to another tube and a volume of 10mM DTT (final concentration) was added to the supernatant and incubated at 56 °C for 1 h in order to reduce any disulfide bonds in proteins. Subsequently, 55 mM IAM (final concentration) was added to each sample to block any cysteines and the solution was incubated for 1 h in the dark. The supernatant was mixed well with a 5× volume of chilled acetone and left to stand for 2 h at −20 °C in order to precipitate the proteins. After centrifugation at 4 °C, 30,000× *g*, the supernatant was discarded, and the pellet was air-dried for 5 min, dissolved in 500 μL 0.5 M TEAB (Applied Biosystems, Milan, Italy), and sonicated at 200 W for 15 min. Lastly, the samples were centrifuged at 4 °C, 30,000× *g* for 15 min. The supernatant was then transferred to a new tube and the protein content was quantified by Bradford as previously reported [82]. A standard protein dilution series was prepared with 0, 2, 4, 6, 8, 10, 12, 14, 16, and 18 μL standard protein (0.2 μg/μL BSA) and 20, 18, 16, 14, 12, 10, 8, 6, 4, 2 μL pure water. After quantification, equal amounts of total protein were separated with sodium dodecyl sulfate polyacrylamide gel electrophoresis (SDS-PAGE) for the assessment of protein quality. Proteins in the supernatant were maintained at −80 °C until further analysis.

### 4.4. iTRAQ Labeling and Strong Cation Exchange

A volume (100 μg) of total protein was removed from each sample solution and digested with Trypsin Gold (Promega, Madison, WI, USA) using a protein/trypsin ratio of 30:1 at 37 °C for 16 h. After digestion with trypsin, the peptides were dried by vacuum centrifugation. Peptides were reconstituted in 0.5 M TEAB and were processed according to the manufacture’s protocol with 8-plex iTRAQ reagent (Applied Biosystems, Foster City, CA, USA). The proteins were labeled with iTRAQ tags as follows: 115 (non-treated 1), 117 (non-treated 2), 118 (IBA-treated 1), and 119 (IBA-treated 2). All of the samples were mixed and then fractionated using an Ultremex SCX (strong cation exchange) column (4.6 × 250 mm) and the Shimadzu LC-20AB HPLC system (Shimadzu, Kyoto, Japan). The peptide samples were reconstituted with 2 mL of mobile phase A (5% ACN pH 9.8) and aspirated at a flow rate gradient of 1 mL/min: 5% mobile phase B (95% ACN, pH 9.8) 10 Min, 5% to 35% mobile phase B 40 min, 35% to 95% mobile phase B 1 min, mobile phase B for 3 min, 5% mobile phase B equilibrated for 10 min. The elution peak was monitored at a wavelength of 214 nm and one fraction was collected each minute. The eluted peptides were pooled into 20 fractions, desalted with a Strata X C18 column (Phenomenex, Torrance, CA, USA), and vacuum-dried.

### 4.5. LC-ESI-MS/MS Analysis

LC-ESI-MS/MS analysis utilized the Triple TOF5600 system (AB SCIEX, Concord, ON, USA) with a Nanospray III source (AB SCIEX, Concord, ON, USA) and a pulled quartz tip emitter (New Objectives, Woburn, MA, USA). The sample fractionation and subsequent liquid chromatography/electrospray ionization tandem mass spectrometry (LC/ESI-MS/MS) analyses were completed using established procedures [83]. Each fraction was resuspended in buffer A (5% ACN, 0.1% FA) and centrifuged at 20,000× *g* for 10 min. On average, the final concentration of peptide was approximately 0.5 μg/μL. 10 µL of supernatant was loaded on a LC-20AD nano-HPLC (Shimadzu, Kyoto, Japan) with an autosampler onto a 2 cm C18 trap column. The peptides were then eluted onto a 10 cm analytical C18 column (inner diameter 75 μm) packed in-house. The samples were loaded at 8 µL/min for 4 min, and the 35 min gradient was subsequently run at 300 nL/min starting from 2 to 35% B (95% ACN, 0.1% FA). This was then followed by a 5 min linear gradient to 60% and subsequently by a 2 min linear gradient to 80%. Maintenance was then conducted at 80% B for 4 min, which was finally followed by a return to 5% in 1 min. Mass spectrometric analysis was conducted in a data-dependent manner utilizing full scans of the Orbitrap mass analyzer (resolution: ≥30,000 at *m/z* 400; automatic gain control: 500,000 ions, Q Exactive TM, Thermo Fisher, Waltham, Massachusetts, USA). The 20 most intense precursor ions were used for mass spectrometer/mass spectrometer (MS/MS) fragmentation and detected at a mass resolution of 17,500 at *m/z* 100 with peptides above a 5-count threshold selected and excluded for 30 s of 30 mDa mass tolerance. The fragmentation was activated with higher energy collision dissociation. Full Fourier transformed mass spectrometry and MS/MS was set to 1 and 0.1 million ions, with a maximum time of accumulation of 2 s. The general workflow of the iTRAQ experiment is presented in Figure S4.

### 4.6. Protein Identification and Functional Annotation

Raw data files acquired from the TripleTOF 5600 System were converted into MGF files using Proteome Discoverer 1.4 (Thermo Scientific), (5600 msconverter) and the MGF files were searched. Proteins identification was performed by using Mascot search engine (Matrix Science, London, UK; version 2.3.02) against the apple genome protein database (Malus x domestica.v3.0.a1 gene set pep.fasta), which contains 93059 sequences. Trypsin was specified as the digesting enzyme; Fragment mass tolerance was 0.05 Da and peptide tolerance mass was 20 ppm; Oxidation (M) and iTRAQ8plex (Y) were variable modifications, while carbamidomethyl (C), iTRAQ8plex (N-term), and iTRAQ8plex (K) were fixed modifications. Each confident protein identification involves at least one unique peptide with a confidence interval ≥95% according to the mascot probability scores. 

Protein quantitation was performed using IQuant software (v2.2.1, BGI-Shenzhen, Shenzhen, China) [84], which integrates the Mascot Percolator [85] algorithm. Simultaneously, a false discovery rate (FDR) analysis was performed using the strategy of Picked protein FDR [86]. Low confidence of peptides with a global FDR ≥1% were removed in the protein analysis. Only peptides that did not have more than one accession number were used for the calculation of the protein ratio. We only used ratios with *p*-values < 0.05, and only fold changes ≥1.2 were considered significant.

Functional annotations of the proteins were conducted using the Blast2GO program against the non-redundant protein database (NR; NCBI: http://www.geneontology.org/). The Cluster of Orthologous Groups of proteins (COG) is a database of direct homologous classification of proteins and was used to predict the possible function of the identified proteins. KEGG (Available online: http://www.genome.jp/kegg/) is a fundamental public database related to biochemical pathways [87]. It was utilized to determine the main biochemical metabolic pathways and signal transduction pathways associated with the identified proteins.

### 4.7. Reverse Transcription-Quantitative PCR (RT-qPCR)

Total RNA was extracted from samples at different time points after the IBA treatment, according to a previously described cetyltrimethylammonium bromide based method with slight modifications [88]. cDNA was reverse transcribed from 1 μg of total RNA using the PrimeScript RT Reagent Kit with gDNA Eraser (TaKaRa Bio, Shiga, Japan), following the manufacturer’s instructions. Relative quantification of gene expression by qPCR was performed on a LightCycler 1.5 instrument (Roche, Mannheim, Germany). The primers used for qPCR were designed using Primer 6 software (Genetyx Software, version 10, Premier Biosoft International, Palo Alto, CA, USA), according to mRNA sequences that were obtained from the Golden genome annotation project (Supplementary Table S5). All qRT-PCR data were normalized using the threshold cycle value for the apple *EF-1a* gene (Table S4). qPCR was performed in an optical 384-well plate, including 10.0 μL SYBR Premix Ex Taq (Takara, Ohtsu, Japan), 0.4 μL primer (10 μM), 2 μL cDNA, and 7.2 μL RNase-free water for a final volume of 20 μL. For the qPCR reactions, the thermocycling conditions were as follows: 95 °C for 30 s; 40 cycles of 95 °C for 5 s and 60 °C for 20 s. The reactions were performed in triplicate and relative gene expression values were calculated using the ddCt algorithm [89]. The results were averaged.

### 4.8. Statistical Analysis

The physiological data were analyzed by one-way ANOVA, followed by two-tailed *t*-test at the 5% level via SPSS statistical software (version 16.0; SPSS, Inc., Chicago, IL, USA). Diagrams were generated in OriginPro 8.0 (OriginLab Software, Inc.,Northampton, MA, USA).

## 5. Conclusions

**Figure 6:**
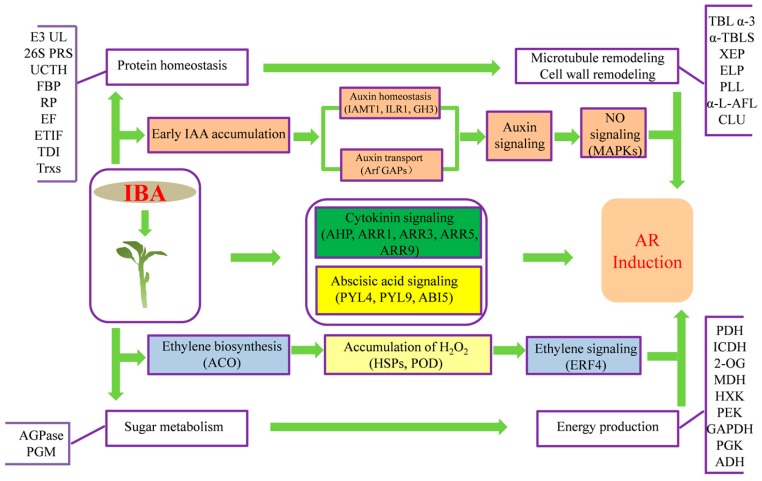
Model of the AR induction pathway in ‘T337’ basal cuttings. After the supplement of exogenous IBA, proteins related to auxin homeostasis and transport facilitated the auxin accumulation in apple, which promoted auxin signaling, as well as nitric oxide signaling. Enhanced ethylene biosynthesis promoted H_2_O_2_ accumulation and ethylene signaling. Meanwhile, cytokinin signal was reduced and abscisic acid signal was enhanced. Sugar metabolism and energy production proteins ensured adequate carbon skeleton and energy supply. Protein homeostasis proteins, especially proteins related to ubiquitinylation might participate in adventitious roots (ARs) formation through the regulation of phytohormone signaling. In addition, microtubule proteins, along with proteins related to cell wall properties, functioned in the shift from cell division to cell differentiation to stimulate AR formation instead of callus formation. IAMT, Indole-3-acetate *O*-methyltransferase 1; ILR1, IAA-amino acid hydrolase; GH3, Indole-3-acetic acid-amido synthetase; Arf GAPs, ADP-ribosylation factor GTPase-activating protein; AHP, Histidine-containing phosphotransfer protein; ARRs, two-component response regulators; PYL9, Abscisic acid receptor PYL9; PYL4, Abscisic acid receptor PYL4; ABI5, ABSCISIC ACID-INSENSITIVE 5-like protein; ACO, 1-aminocyclopropane-1-carboxylate oxidases; HSPs, Heat shock 70 kDa protein; POD, Peroxidase; MAPK, Mitogen-activated protein kinase; ERF4, Ethylene-responsive transcription factor 4; E3 UL, E3 ubiquitin ligase; 26S PRS, 26S proteasome regulatory subunits; UCTH, Ubiquitin C-terminal hydrolases; FBP, F-box protein; RP, Ribosomal protein; EF, Elongation factor; ETIF, Eukaryotic translation initiation factor; TDI, Thiol-disulfide isomerase; Trxs, Thioredoxins; TBL α-3, Tubulin alpha-3; α-TBLS, Alpha-tubulin suppressor; XEP, Xyloglucan endotransglucosylase protein; ELP, Expansin-like protein; PLL, Pectin lyase-like superfamily protein; α-l-AFL, α-l-arabinofuranosidase; CLU, Cellulase; AGPase, ADP-glucose pyrophosphorylase; PGM, phosphoglucomutase; PDH, Pyruvate dehydrogenase; ICDH, Isocitrate dehydrogenase; 2-OG, 2-oxoglutarate; MDH, malic enzyme; HXK, Hexokinase; PEK, 6-phosphofructokinase; GAPDH, glyceraldehyde-3-phosphate dehydrogenase; PGK, 3-phosphoglycerate kinase; ADH, alcohol dehydrogenase.

An iTRAQ-based proteomic approach was employed to compare the abundance of proteins between untreated and IBA-treated apple rootstock cuttings at 3 day after treatment. The regulation and expression of proteins involved in a wide range of processes were revealed. A complex phytohormone signal transduction mechanism plays a crucial role in IBA-induced AR formation. Specific enzymes and metabolites participating in carbohydrate process, TCA cycle and glycolysis may significantly enhance carbon skeleton supply and energy production. Changes were also identified in ubiquitinylation, ROS, and NO signaling, and cell wall remodeling. Finally, we constructed a putative model to illustrate the underlying molecular mechanisms that are associated with IBA-mediated AR induction in ‘T337’apple rootstocks (Figure 6). The current study provides further information for understanding the processes of adventitious root formation in apple rootstocks, as well as other fruit trees.

## Figures and Tables

**Table 1:** Kyoto Encyclopedia of Genes and Genomes (KEGG) Pathway Enrichment Analysis of differentially expressed proteins (DEPs) (*p* < 0.05).

Pathway	DEPs (2868) *^a^*	All Proteins (6476) *^b^*	Pathway ID	*p*-Value *^c^*
Metabolic pathways	873 (30.44%)	1845 (28.49%)	ko01100	0.001077
Biosynthesis of secondary metabolites	505 (17.61%)	1067 (16.48%)	ko01110	0.01567
Starch and sucrose metabolism	108 (3.77%)	216 (3.34%)	ko00500	0.049829
Ribosome	135 (4.71%)	237 (3.66%)	ko03010	0.0000442
Phenylpropanoid biosynthesis	91 (3.17%)	140 (2.16%)	ko00940	0.000000492
Pentose and glucuronate interconversions	77 (2.68%)	142 (2.19%)	ko00040	0.010236
Peroxisome	68 (2.37%)	121 (1.87%)	ko04146	0.005217
Carbon fixation in photosynthetic organisms	56 (1.95%)	104 (1.61%)	ko00710	0.030497
Glycine, serine and threonine metabolism	51 (1.78%)	87 (1.34%)	ko00260	0.00477
Glutathione metabolism	50 (1.74%)	85 (1.31%)	ko00480	0.004702
Glyoxylate and dicarboxylate metabolism	40 (1.39%)	68 (1.05%)	ko00630	0.010849
Cyanoamino acid metabolism	40 (1.39%)	70 (1.08%)	ko00460	0.020206
beta-Alanine metabolism	34 (1.19%)	52 (0.8%)	ko00410	0.001692
Phenylalanine metabolism	34 (1.19%)	53 (0.82%)	ko00360	0.00273
Flavonoid biosynthesis	34 (1.19%)	54 (0.83%)	ko00941	0.004267
Other glycan degradation	34 (1.19%)	57 (0.88%)	ko00511	0.013751
Fatty acid degradation	30 (1.05%)	53 (0.82%)	ko00071	0.047544
alpha-Linolenic acid metabolism	29 (1.01%)	49 (0.76%)	ko00592	0.025178
Terpenoid backbone biosynthesis	29 (1.01%)	50 (0.77%)	ko00900	0.035041
Photosynthesis	27 (0.94%)	42 (0.65%)	ko00195	0.00701
Ubiquinone and other terpenoid-quinone biosynthesis	25 (0.87%)	39 (0.6%)	ko00130	0.009848
Sphingolipid metabolism	25 (0.87%)	42 (0.65%)	ko00600	0.033381
Tropane, piperidine and pyridine alkaloid biosynthesis	21 (0.73%)	31 (0.48%)	ko00960	0.007088
Stilbenoid, diarylheptanoid and gingerol biosynthesis	20 (0.7%)	29 (0.45%)	ko00945	0.006307
Limonene and pinene degradation	17 (0.59%)	20 (0.31%)	ko00903	0.000214
Lysine degradation	17 (0.59%)	26 (0.4%)	ko00310	0.024492
Photosynthesis—antenna proteins	7 (0.24%)	8 (0.12%)	ko00196	0.016319
Phenylpropanoid biosynthesis	91 (3.17%)	140 (2.16%)	ko00940	0.000000492

*^a^* Differentially expressed proteins (DEPs) were analyzed with pathway annotation; *^b^* All proteins were analyzed with pathway annotation; *^c^* Pathways with *p*-value higher than 0.05 were not listed.

**Table 2:** List of proteins differently expressed in ‘T337’ basal cuttings with IBA treatment and control.

Accession No. *^a^*	Description	SwissProt Accession *^b^*	Identity (%) *^c^*	%COV (95) *^d^*	Unique Peptides	Ratio *^e^*
**Phytohormone signaling**						
MDP0000873893	Indole-3-acetic acid-amido synthetase	O82333	80.27	5.7	1	2.03
MDP0000121609	Indole-3-acetic acid-amido synthetase	O82333	80.14	17	2	1.64
MDP0000310711	IAA-amino acid hydrolase ILR1	P54968	54.53	4.2	2	1.74
MDP0000663451	IAA-amino acid hydrolase ILR1	P54968	62.83	10	3	1.52
MDP0000290695	Indole-3-acetate O-methyltransferase 1	Q9FLN8	73.58	1.8	1	0.43
MDP0000140463	ADP-ribosylation factor gtpase-activating protein	O80925	70.28	6.8	3	0.67
MDP0000250432	ADP-ribosylation factor gtpase-activating protein	O80925	54.84	1.4	1	0.58
MDP0000288128	ADP-ribosylation factor gtpase-activating protein	O80925	62.68	1.3	1	0.37
MDP0000186518	Histidine-containing phosphotransfer protein 3	Q9SAZ5	56.1	6.1	1	0.32
MDP0000509768	Arabidopsis response regulator ARR9	O80366	59.18	5.7	1	0.45
MDP0000212178	Arabidopsis response regulator ARR5	Q9ZWS6	78.01	4	1	0.53
MDP0000250737	Arabidopsis response regulator ARR3	Q9ZWS9	76.61	5.9	2	0.29
MDP0000846313	Arabidopsis response regulator ARR1	Q940D0	63.64	5.7	1	0.65
MDP0000175691	1-aminocyclopropane-1-carboxylate oxidase 1	Q9LSW7	29.34	6	1	1.57
MDP0000663852	1-aminocyclopropane-1-carboxylate oxidase 1	Q0WPW4	46.59	4.8	1	1.6
MDP0000839921	1-aminocyclopropane-1-carboxylate oxidase 1	Q84MB3	47.74	7	2	1.81
MDP0000195885	1-aminocyclopropane-1-carboxylate oxidase 1	Q00985	100	7.6	2	0.29
MDP0000324718	Ethylene-responsive transcription factor 4	O80340	74.71	5.6	1	0.57
MDP0000284624	Abscisic acid receptor PYL9	Q84MC7	81.29	6	1	1.56
MDP0000215106	Abscisic acid-insensitive 5-like protein 2	Q9LES3	52.22	3.2	1	0.51
MDP0000228470	Abscisic acid receptor PYL4	O80920	80.12	4.6	1	0.58
**Carbohydrate metabolism and energy production**						
MDP0000866748	Phosphoglucomutase	Q9SCY0	84.31	14.9	7	1.79
MDP0000256619	ADP-glucose pyrophosphorylase 1	P52417	88.72	16.7	1	2.44
MDP0000298815	α-amylase	P17859	72.8	2.2	2	2.2
MDP0000657082	α-mannosidase	P34098	61.35	9.1	2	1.84
MDP0000095637	Granule-bound starch synthase 1	O82627	74.56	21.2	9	2.69
MDP0000133306	d-sorbitol-6-phosphate dehydrogenase	P28475	84.52	11.9	1	2.62
MDP0000661960	Xyloglucan endotransglucosylase/hydrolase protein 6	Q8LF99	78.36	14.4	2	2.7
MDP0000129346	α-1,4 glucan phosphorylase L isozyme	P53536	81.22	21.4	15	1.63
MDP0000320017	Xyloglucan endotransglucosylase/hydrolase protein	Q38910	75.76	2.8	1	1.5
MDP0000296747	α-glucosidase	Q9F234	45.19	8.7	5	1.5
MDP0000177786	1,4-α-glucan-branching enzyme	P30924	73.7	10.7	7	1.5
MDP0000202465	β-galactosidase	Q9FN08	60.98	19	13	0.19
MDP0000290090	β-galactosidase 6	Q10NX8	42.16	4.8	1	0.47
MDP0000295518	α-l-fucosidase 1	Q8GW72	71.43	10.7	4	1.67
MDP0000863563	β-galactosidase 9	Q9SCV3	77.39	16.6	5	1.71
MDP0000237069	α-galactosidase	Q9FXT4	25.52	8.4	2	1.71
MDP0000823956	Hexokinase	Q9SEK2	78.16	13.7	3	1.65
MDP0000294262	6-phosphofructokinase	Q41141	82.18	8.1	1	0.4
MDP0000254412	6-phosphofructokinase	Q8VYN6	79.88	5.5	1	1.55
MDP0000835914	Glyceraldehyde-3-phosphate dehydrogenase	P12859	88.47	16.2	5	1.62
MDP0000527995	Glyceraldehyde-3-phosphate dehydrogenase	P12858	88.15	24.8	6	1.69
MDP0000543856	Glyceraldehyde-3-phosphate dehydrogenase	Q8S0G4	79.87	6.5	1	1.84
MDP0000174843	3-phosphoglycerate kinase	Q42961	88.13	22.2	1	1.63
MDP0000212948	3-phosphoglycerate kinase	Q42961	84.14	23.8	1	0.57
MDP0000211987	Aldehyde dehydrogenase	Q9ZPB7	82.41	8.7	4	1.57
MDP0000221713	Aldehyde dehydrogenase	Q9SU63	82.05	18.4	7	2
MDP0000236430	Alcohol dehydrogenases	P42734	66.77	8.6	4	1.78
MDP0000267169	Fructose-1,6-bisphosphatase	P46283	75.31	4.9	1	2.07
MDP0000273014	Fructose-1,6-bisphosphatase	P46275	78.87	19.5	6	1.67
MDP0000244771	Fructose-1,6-bisphosphatase	P46283	83.72	5.8	3	1.57
MDP0000277811	Fructose-1,6-bisphosphatase	P46283	80.67	5	1	1.95
MDP0000275261	Probable fructokinase-2	Q9LNE3	65.8	8.5	1	1.57
MDP0000178814	Pyruvate dehydrogenase complex	Q54M22	53.72	6.3	1	1.59
MDP0000325085	Isocitrate dehydrogenase	P50217	89.78	21.1	3	1.54
MDP0000214399	2-oxoglutarate and Fe(II)-dependent oxygenase superfamily protein	Q39110	26.8	3.2	1	1.68
MDP0000384593	Malic enzyme	P12628	83.78	23.7	8	1.58
MDP0000221561	Malic enzyme	P51615	83.94	24.4	7	1.5
MDP0000313179	NADH: ubiquinone oxidoreductase 20 kd subunit	P42027	98.11	5.5	1	1.53
MDP0000362465	Cytochrome b-c1 complex subunit 9	P46270	76.39	22.4	2	1.57
MDP0000124616	Cytochrome c oxidase subunit 5C-2	Q9LZQ0	77.78	12.7	1	1.65
MDP0000263444	Cytochrome c oxidase subunit 6b-1	Q9S7L9	84.06	4.5	1	0.41
MDP0000385730	ATP synthase gamma chain, chloroplastic	P29790	85.68	9.7	3	1.75
MDP0000168167	Atpases with chaperone activity, chloroplastic	Q9LF37	81.33	16.6	2	1.73
MDP0000929055	ATP synthase subunit alpha, mitochondrial	P05495	99.16	10.1	2	1.65
MDP0000360515	ATP synthase subunit beta, mitochondrial	P17614	93.16	38.9	2	1.59
MDP0000785964	ATP synthase subunit delta, mitochondrial	Q40089	92.11	13.9	1	0.41
MDP0000624197	ATP synthase subunit delta, mitochondrial	Q40089	78.5	15.4	3	0.39
MDP0000416290	Atpase 4, plasma membrane-type	Q9SU58	84.62	5.5	1	0.58
**ROS and NO signaling**						
MDP0000684170	Heat shock 70 kda protein	Q9SKY8	69.76	3.3	1	1.54
MDP0000697285	Heat shock 70 kda protein	Q02028	79.23	12.4	1	1.64
MDP0000319048	Cationic peroxidase 1	P22195	74.6	21.3	6	0.43
MDP0000545323	Peroxidase 42	Q9SB81	83.55	8.4	1	0.47
MDP0000243237	Peroxidase 44	Q93V93	56.11	33.5	7	0.35
MDP0000208152	Peroxidase 53	Q42578	60.98	9.8	2	0.43
MDP0000154541	Peroxidase 53	Q42578	63.5	13.2	1	0.66
MDP0000192235	Peroxidase 42	Q9SB81	75.44	6.1	1	0.67
MDP0000706473	Peroxidase 53	Q42578	63.61	17.4	2	0.67
MDP0000283650	Peroxidase 4	A7NY33	62.46	7	2	0.62
MDP0000136398	Peroxidase 16	Q96518	64.42	4.6	1	0.49
MDP0000301828	Peroxidase 53	Q42578	79.22	12.7	2	0.49
MDP0000209189	Cationic peroxidase 1	P22195	73.58	29	5	0.55
MDP0000251955	Mitogen-activated protein kinase	Q40353	86.38	5.9	2	1.51
**Protein homeostasis**						
MDP0000241084	E3 ubiquitin-protein ligase RGLG1	Q9SS90	71.97	6.7	1	1.51
MDP0000317971	E3 ubiquitin-protein ligase UPL2	Q8H0T4	48.73	3.9	3	0.57
MDP0000269081	E3 ubiquitin-protein ligase RING1	P0CH30	58.54	3.4	1	0.64
MDP0000676693	ATP-dependent 26S proteasome regulatory subunit	Q54DY9	34	22.7	4	2.03
MDP0000322270	ATP-dependent 26S proteasome regulatory subunit	Q7ZZ25	42.23	6.2	1	0.58
MDP0000315993	ATP-dependent Zn proteases	O80860	85.11	4.7	6	1.61
MDP0000188831	20S proteasome, alpha and beta subunits	O82178	58.71	16.2	5	0.45
MDP0000245541	Ubiquitin carboxyl-terminal hydrolase 2	Q8W4N3	51.36	0.9	1	0.45
MDP0000283283	Ubiquitin carboxyl-terminal hydrolase 24	Q9FPS3	74.27	5.8	2	0.64
MDP0000263256	F-box protein	Q9FGY4	28.24	1.7	1	1.73
MDP0000180936	F-box protein	Q9FE83	54.18	4.9	1	2.38
MDP0000141860	40S ribosomal protein S30	P49689	98.39	16.1	1	0.38
MDP0000265859	60S ribosomal protein L14-1	Q9SIM4	87.72	14.7	2	0.53
MDP0000865687	40S ribosomal protein S17-4	Q9LZ17	83.69	17.9	1	0.39
MDP0000544199	50S ribosomal protein L24	A8LC45	49.51	10.9	1	0.39
MDP0000283097	40S ribosomal protein S17-4	Q9LZ17	79.43	7.3	1	0.45
MDP0000266765	30S ribosomal protein S5	P93014	68.2	9.5	3	0.64
MDP0000772832	40S ribosomal protein S19-3	Q9FNP8	78.06	25.2	2	0.58
MDP0000417422	40S ribosomal protein S5	O24111	90.24	18.7	1	0.59
MDP0000169133	Translation elongation factor	B7K735	62.8	2.6	2	0.44
MDP0000903484	Translation elongation factor EF-1	P93447	62.83	21.5	2	0.44
MDP0000800338	Translation initiation factor IF-2	P57997	70.64	5.5	1	0.62
MDP0000142167	Eukaryotic translation initiation factor 2 subunit	P55871	96.68	7.5	1	0.63
MDP0000270113	Eukaryotic translation initiation factor 3 subunit	Q40554	64.54	9.5	2	0.6
MDP0000141898	Eukaryotic translation initiation factor 3 subunit	Q38884	81.19	14.4	1	0.67
MDP0000261642	Tryptophanyl-tRNA ligase	Q5UPJ7	44.83	3.1	2	1.63
MDP0000134153	Histidine–tRNA ligase	P93422	33.14	2	1	1.61
MDP0000770881	Glycine–tRNA ligase 1	O23627	58.43	8.5	1	1.56
MDP0000207727	Thiol-disulfide isomerase and thioredoxins	O80763	64.64	20.8	3	2.63
MDP0000308890	Thiol-disulfide isomerase and thioredoxins	O80763	62.36	15.5	3	2.39
MDP0000297301	Thiol-disulfide isomerase and thioredoxins	O80763	59.55	2.2	1	1.81
MDP0000505556	Cysteine protease	P25776	44.1	3.1	1	1.77
**Microtubules and Cell wall remolding**						
MDP0000812416	Tubulin α-3	P33627	96.05	18.3	2	1.58
MDP0000282827	α-tubulin suppressor	Q9P258	77.58	3.9	2	0.67
MDP0000296747	Xyloglucan endotransglucosylase/hydrolase	Q9F234	45.19	8.7	5	1.5
MDP0000661960	Xyloglucan endotransglucosylase/hydrolase	Q8LF99	78.36	14.4	2	2.7
MDP0000640549	Expansin-like protein	O23547	54.84	5.9	1	2.16
MDP0000130769	Pectin lyase-like superfamily protein	A7PZL3	52.81	12.5	4	1.53
MDP0000248311	Pectin lyase-like superfamily protein	P15922	26.05	5.2	3	1.72
MDP0000943790	Pectin lyase-like superfamily protein	Q949Z1	47.68	1.9	1	0.3
MDP0000175757	Pectin lyase-like superfamily protein	Q94AJ5	45.06	4.2	2	0.49
MDP0000251956	Pectin lyase-like superfamily protein	A7PZL3	80.32	17.4	4	0.49
MDP0000753366	Cellulase	P23548	24.41	4.8	1	2.32
MDP0000055078	α-l-arabinofuranosidase 1	Q9SG80	70.21	16.8	5	1.57

*^a^* Accession no. is the locus name of a gene in apple genome; *^b^* SwissProt Accession is matched accession of blast in SwissProt database; *^c^* Identity means the score of blast in SwissProt database; *^d^* %COV (95) indicates the percentage of matching amino acids from identified peptides having confidence greater than or equal to 95%; *^e^* Mean ratio corresponds to protein reporter ion intensity originating from IBA-treated protein samples relative to control protein samples with a 1.5-fold change and a *p* < 0.05.

## Supplementary Materials

The following are available online at http://www.mdpi.com/1422-0067/19/3/667/s1.

## Abbreviations

iTRAQ	Isobaric tags for relative and absolute quantification
AR	Adventitious root
LR	Lateral root
IBA	Indole-3-butyric acid
IAA	Indole-3-acetic acid
CK	Cytokinin
ABA	Abscisic acid
GA	Gibberellin
JA	Jasmonic acid
BR	Brassinolide
HPLC	High Performance Liquid Chromatography
CHAPS	3-[(3-Cholamidopropyl) dimethylammonio]propanesulfo
PMSF	Phenylmethylsulfonyl fluoride
EDTA	Ethylene Diamine Tetraacetic Acid
MS	Murashige and Skoog salts
TCA	Trichloroacetic Acid
DTT	dl-Dithiothreitol
IAM	Iodoacetamide
TEAB	Tetraethyl-ammonium bromide
ACN	Acetonitrile
BSA	Bull Serum Albumin
FA	Formic Acid
FDR	False Discovery Rate
NP40	Nonidet P-40
